# The magic triangle goes MAD: experimental phasing with a bromine derivative

**DOI:** 10.1107/S0907444909051609

**Published:** 2010-03-24

**Authors:** Tobias Beck, Tim Gruene, George M. Sheldrick

**Affiliations:** a Department of Structural Chemistry, Georg-August-Universität Göttingen, Tammannstrasse 4, 37077 Göttingen, Germany

**Keywords:** multi-wavelength anomalous dispersion, experimental phasing, heavy-atom derivatives

## Abstract

Experimental phasing is an essential technique for the solution of macromolecular structures. Since many heavy-atom ion soaks suffer from nonspecific binding, a novel class of compounds has been developed that combines heavy atoms with functional groups for binding to proteins. The phasing tool 5-amino-2,4,6-tribromoisophthalic acid (B3C) contains three functional groups (two carboxylate groups and one amino group) that interact with proteins *via* hydrogen bonds. Three Br atoms suitable for anomalous dispersion phasing are arranged in an equilateral triangle and are thus readily identified in the heavy-atom substructure. B3C was incorporated into proteinase K and a multiwavelength anomalous dispersion (MAD) experiment at the Br *K* edge was successfully carried out. Radiation damage to the bromine–carbon bond was investigated. A comparison with the phasing tool I3C that contains three I atoms for single-wavelength anomalous dispersion (SAD) phasing was also carried out.

## Abstract 2

5-Amino-2,4,6-tribromoisophthalic acid is used as a phasing tool for protein structure determination by MAD phasing. It is the second representative of a novel class of compounds for heavy-atom derivatization that combine heavy atoms with amino and carboxyl groups for binding to proteins.

## 1. Introduction   

Experimental phasing is vital for the determination of three-dimensional protein structures using single-crystal X-ray diffraction. Although about two-thirds of newly deposited structures in the Protein Data Bank were solved using molecular replacement, experimental phasing suffers less from model bias and is required for samples that do not have any structurally related entries.

Methods for experimental phasing based on the anomalous scattering of certain atoms, SAD (single-wavelength anomalous dispersion) and MAD (multi-wavelength anomalous dispersion), have largely replaced traditional methods such as MIR (multiple isomorphous replacement).^1^ With the latest advances in synchrotron hardware, *e.g.* improved detectors, phasing with the weak anomalous signal from intrinsic scatterers has become a possible option, although high-quality data are required. These are more readily obtained when high symmetry enables a high data redundancy to be achieved, but low-symmetry examples have also proved successful (Lako­mek *et al.*, 2009 ▶).

In the case of suboptimal data quality or the absence of suitable intrinsic scatterers, external anomalously scattering atoms have to be introduced into the protein crystal. The main route for solving novel protein structures is without doubt the use of a selenomethionine derivative (Hendrickson, 1999 ▶). Similarly, the chemical incorporation of brominated nucleobases has become an important technique for nucleic acid structure determination (Peterson *et al.*, 1996 ▶). Heavy-atom soaks traditionally have a low success rate, but systematic heavy-atom screening with conventional heavy-metal ions has been performed using gel electrophoresis (Boggon & Shapiro, 2000 ▶), mass spectrometry (Agniswamy *et al.*, 2008 ▶) or a database approach (Sugahara *et al.*, 2005 ▶).

It has also been shown that a more comprehensive treatment of anomalous scattering can yield further phase information. The anisotropy of anomalous scattering can be exploited to enhance the phase information present in the collected data (Schiltz & Bricogne, 2008 ▶). The combination of phase information from a partial molecular-replacement solution and weak experimental phases has also led to a number of successes (*e.g.* Tereshko *et al.*, 2008 ▶; Schuermann & Tanner, 2003 ▶; Roversi *et al.*, 2010 ▶), and is also included in the Auto-Rickshaw server (Panjikar *et al.*, 2009 ▶).

Since most of these are specialized applications and are not generally applicable, a quick and easy approach towards experimental phasing would still be desirable for the deriv­atization of protein crystals and experimental phase determination.

## 2. The magic triangle I3C   

Often, heavy-atom derivatives suffer from nonspecific binding. This results in low occupancy of the heavy-atom sites or in derivatization failing completely. We have developed a new class of compounds that combine heavy atoms for phasing with functional groups for specific interaction with biological macromolecules.

**Figure 1:**
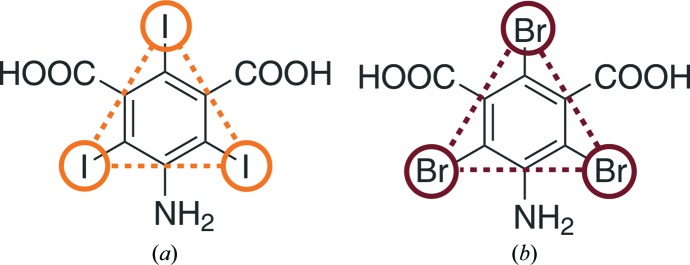
The phasing tools I3C and B3C. (*a*) The magic triangle I3C (5-amino-2,4,6-triiodoisophthalic acid) with the equilateral triangle of I atoms (side of 6.0 Å) shown. (*b*) The MAD triangle B3C (5-amino-2,4,6-tribromo­isophthalic acid) with the equilateral triangle of Br atoms (side of 5.65 Å) shown.

The first representative of this novel class of compounds is 5-­amino-2,4,6-triiodoisophthalic acid (hereafter referred to as I3C; Fig. 1 ▶ *a*). The three I atoms, which are arranged in an equilateral triangle (with a side of 6 Å), give rise to a strong anomalous signal using in-house Cu *K*α radiation. I3C has been incorporated into three test proteins (lysozyme, thaumatin and porcine elastase) either by cocrystallization or soaking (Beck *et al.*, 2008 ▶). The three functional groups of I3C interact with the protein *via* hydrogen bonds. The amino group interacts with hydrogen acceptors such as the carbonyl O atom of asparagine or glutamine residues and, most importantly, with the carbonyl O atom of the protein backbone. The carboxylate groups interact with the hydrogen-donor groups found in serine, threonine, lysine or tyrosine. The most pro­minent interaction of the carboxylate group is its inter­action with arginine. Since the three I atoms in I3C form an equilateral triangle, a successful substructure solution can readily be identified when inspecting the heavy-atom positions.

I3C has also been used to solve a novel protein structure. Experimental phases could be derived for the 35 kDa protein Mh-p37, which had resisted other phasing attempts (Sippel *et al.*, 2008 ▶). I3C was introduced by soaking the protein crystals at a low pH value (in contrast to pH values of 6–8 for the test proteins in Beck *et al.*, 2008 ▶). The derivatization was successful using a lower I3C concentration (40 m*M* instead of 0.25 or 0.5 *M* as for the test proteins).

The strong anomalous signal of the I atoms renders I3C a powerful phasing tool for both in-house and synchrotron data. However, the I *K* edge (λ = 0.374 Å) and *L*_I_ edge (λ = 2.39 Å) are not commonly accessible on synchrotron beamlines. Therefore, I3C can only be used for SAD or SIRAS phasing.

## 3. The MAD triangle B3C   

Here, we report on the new phasing tool 5-amino-2,4,6-tri­bromoisophthalic acid (hereafter referred to as B3C; Fig. 1 ▶ *b*). Analogously to I3C, the bromine compound B3C has three functional groups for hydrogen bonding. The three Br atoms arranged in an equilateral triangle (with a side of 5.65 Å) are suitable for MAD experiments since the Br *K* edge (λ = 0.920 Å) falls within the normal energy range of a macromolecular crystallography beamline.

B3C was incorporated into crystals of proteinase K and a four-wavelength MAD experiment was carried out. Radiation damage was also investigated since it is known that radiolysis of the anomalous scatterers, *e.g.* the Br atom in brominated nucleotides, can prevent structure solution (Ennifar *et al.*, 2002 ▶). We also compared the phasing ability of B3C with that of I3C for proteinase K.

## 4. Methods   

### 4.1. Crystallization and protein derivatization   

In this study, the incorporation of B3C and I3C into proteinase K was investigated. Synthesis and crystallographic characterization of B3C have been described elsewhere (Beck, Herbst-Irmer *et al.*, 2009 ▶). Practical advice on the incorporation of B3C (and I3C) into protein crystals is also available (Beck, da Cunha *et al.*, 2009 ▶). Stock solutions of B3C and I3C with 1 *M* concentration were obtained by dissolving the solid materials in 2 *M* lithium hydroxide solution to deprotonate the two carboxyl groups.^2^ Protein crystals were obtained using the sitting-drop vapour-diffusion method.

Proteinase K (279 residues, 28.9 kDa; obtained from Sigma–Aldrich) was crystallized at 293 K by mixing 2.5 µl 20 mg ml^−1^ protein solution with an equal volume of precipitant containing 0.1 *M* Tris pH 7.2 and 1.28 *M* ammonium sulfate. Crystals appeared within one week. Protein crystals were soaked for about 10 s in 0.5 *M* B3C or I3C solution which also contained the same salt and buffer concentrations as the crystallization drop. The crystals were back-soaked for 5 s in a cryosolution containing the same salt and buffer concentrations with 30% glycerol but no heavy-atom compound. The crystals were then flash-cooled in liquid nitrogen.

### 4.2. Data collection and processing   

**Figure 2:**
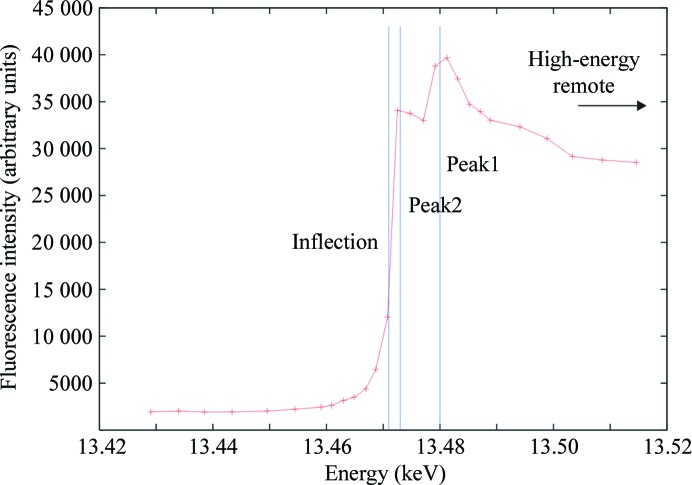
Fluorescence scan of proteinase K with B3C incorporated. The data-collection energies of peak1, peak2 and the inflection point (see Table 1 ▶) are marked by vertical lines. The two peaks close to the Br *K* edge are clearly visible.

Data were collected at 106 K on beamline X10SA (PXII) at SLS, Villigen, Switzerland. For the B3C data, a fluorescence scan was performed to locate the Br *K* edge. Interestingly, the spectrum showed two peaks at the Br edge (Fig. 2 ▶). Therefore, two peak data sets were collected, followed by high-energy remote and inflection-point data sets (see Table 1 ▶ for details). For the I3C data only one data set was collected, at the same wavelength as in-house Cu *K*α (1.54178 Å; Table 1 ▶). Data collection for all data sets was carried out with 0.5 s exposure per frame and 1° frame width.

To investigate radiation damage to B3C, a series of experiments was carried out. The same crystal that had been used for the MAD experiment was exposed to full photon flux (1.82 × 10^12^ photons s^−1^ at 13.473 keV), followed by data collection at the same energy (corresponding to the wavelength at peak2; Table 1 ▶). Full beam exposure was maintained for 2 s and was increased to 30 and 60 s for the last two burn exposures, respectively. A total of six data sets were collected from the same crystal following this procedure.

Data were integrated with *XDS* (Kabsch, 1993 ▶). Absorption correction and scaling were carried out with *SADABS* (Sheldrick, 1996 ▶).

## 5. Results and discussion   

### 5.1. Substructure solution and data analysis   

**Figure 3:**
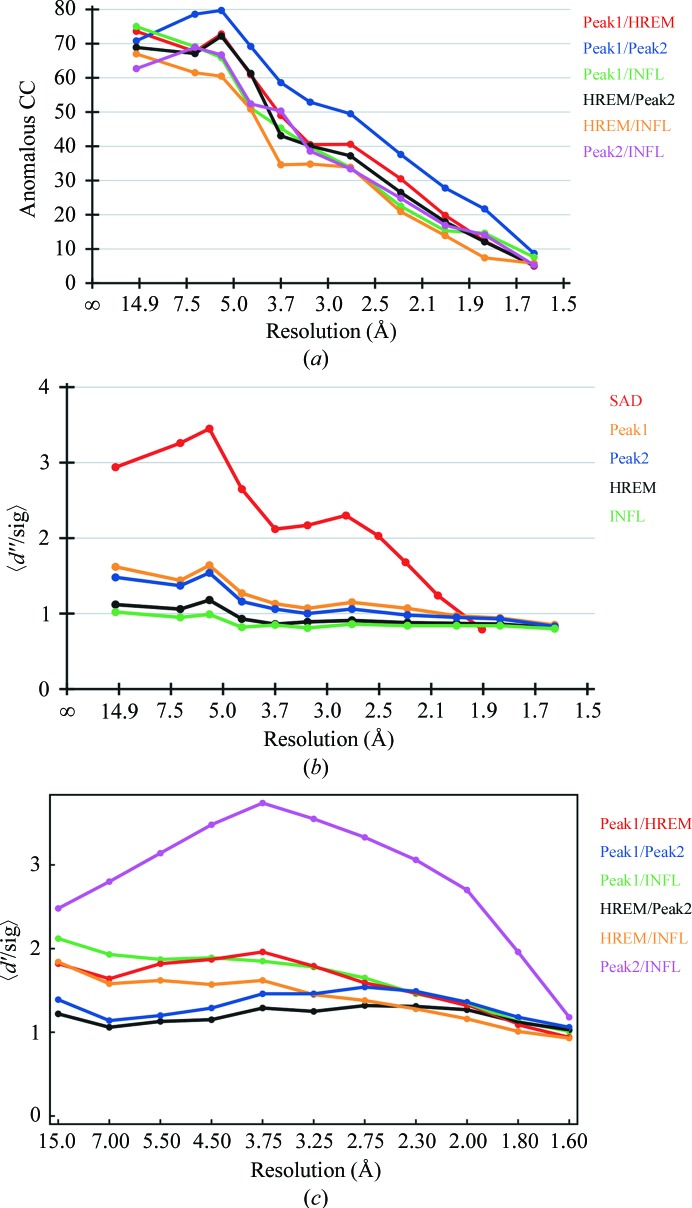
Anomalous and dispersive data statistics. (*a*) Anomalous correlation coefficient (CC) between the MAD data sets. HREM, high-energy remote; INFL, inflection point (data-collection wavelengths can be found in Table 1 ▶). The correlation coefficients do not depend on the (estimated) σ values. Data were truncated at 2.5 Å for heavy-atom substructure solution (where CC falls below 30% for all data sets). (*b*) Anomalous signal for the four bromine MAD data sets and the iodine SAD data set (red); pure noise would correspond to *d*′′/sig ≃ 0.798. Here, the cutoff for the MAD data sets cannot be determined easily. The iodine data set shows a strong anomalous signal (data collected at 1.5418 Å). Data were truncated at 2.0 Å for heavy-atom substructure solution for the iodine data set (where *d*′′/sig falls below 1.2). (*c*) Dispersive differences between the MAD data sets. HREM, high-energy remote; INFL, inflection point (data-collection wavelengths can be found in Table 1 ▶). Radiation damage could have caused the apparent differences in *d*′ values; because one wavelength was completed before the next wavelength was measured, systematic errors could be introduced that could also lead to an overestimation of the dispersive signal.

For B3C, four data sets (two peak, one high-energy remote and one inflection point) were used for MAD phasing (see Fig. 3 ▶ for anomalous data statistics). Data sets were prepared with *XPREP* (Bruker AXS Inc., Madison, Wisconsin, USA). Substructure solution was carried out with *SHELXD* (Schneider & Sheldrick, 2002 ▶). Inspection of the heavy-atom sites revealed the presence of equilateral triangles (with side lengths of about 5.6 Å).

The two peaks in the fluorescence spectrum (Fig. 2 ▶) can be rationalized by the anisotropy of the anomalous signal, although fluorescence scans at different orientations of the crystal (not carried out) would be required to confirm this. Similar effects have been observed for proteins containing selenomethionine and brominated nucleotides (Schiltz & Bricogne, 2008 ▶).

For I3C, SAD phasing was carried out using data collected at 1.54178 Å (*f*′′ for iodine is 6.85 e at this wavelength; see Fig. 3 ▶ *b* for anomalous data statistics). Substructure solution with *SHELXD* resulted in heavy-atom sites that also formed equilateral triangles (with a side length of about 6 Å).

Density modification was carried out with *SHELXE* (Sheldrick, 2002 ▶). Model building was performed with *ARP*/*wARP* (Perrakis *et al.*, 1999 ▶), *REFMAC* (Murshudov *et al.*, 1997 ▶) and *Coot* (Emsley & Cowtan, 2004 ▶). Refinement was carried out with the *SHELX* suite (Table 2 ▶; Sheldrick, 2008 ▶). Stereochemical analysis of the refined structures was per­formed with the *MolProbity* server (Davis *et al.*, 2007 ▶). Figures were prepared using *PyMOL* (DeLano, 2008 ▶). Fig. 3 was prepared using *HKL*2*MAP* (Pape & Schneider, 2004 ▶).

Free variables were introduced to refine the occupancy of each B3C or I3C site and subsequently also the occupancy of each single halogen atom separately in B3C or I3C. For the occupancy refinement, thermal displacement parameters were kept fixed for the halogen atoms (at *B* = 15.8 Å^2^ for both bromine and iodine). The final model from the MAD data-set refinement (peak2) was used for further refinement of the radiation-damage data sets.

A comparison of the results from the MAD and the SAD phasing experiments can be found in Table 3 ▶.

### 5.2. B3C and I3C binding sites   

**Figure 4:**
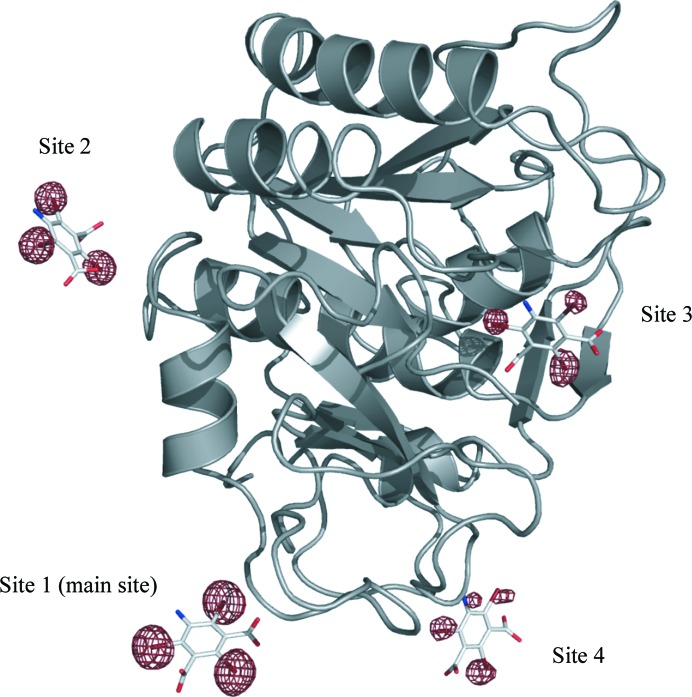
B3C in proteinase K: substructure density calculated with *SHELXE* (using *F*_A_ and α derived from the dispersive and anomalous differences) is contoured at 4σ and covers the whole asymmetric unit. Clear density for the four B3C molecules can be seen.

**Figure 5:**
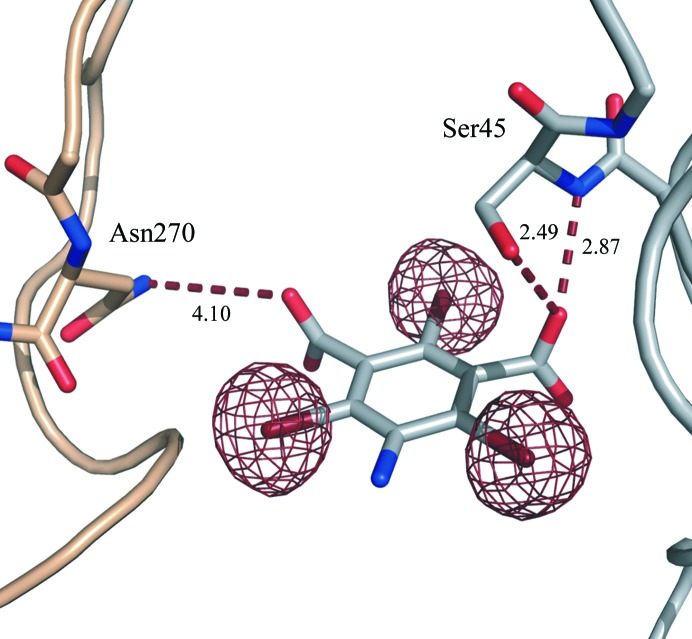
B3C (site 1) in proteinase K at the interface of two proteinase K molecules. Substructure density calculated with *SHELXE* (using *F*_A_ and α) is shown for B3C at 4σ. Hydrogen bonds are depicted as dashed lines and distances are given in Å. The two carboxylate groups interact with Asn270 and Ser45.

Four binding sites for B3C (Fig. 4 ▶) and three binding sites for I3C were observed in proteinase K. Two sites coincide for both derivatives; one of these is the main site with the highest occupancy shown in Fig. 5 ▶. The common occupancies for all three halogen atoms per site were refined with *SHELXL* to 0.42, 0.13, 0.10 and 0.09 for B3C (refinement against peak2 data set)^3^ and 0.72, 0.17 and 0.14 for I3C. Interestingly, the occupancy of the main site differs significantly although similar soaking conditions were used. The difference might be a consequence of different soaking times or crystal properties or may be attributed to the different chemical properties of the two compounds (containing either I or Br atoms).

The interactions of the small molecules in proteinase K are very similar to those previously reported for lysozyme, thaumatin and elastase (Beck *et al.*, 2008 ▶). The three functional groups of the phasing tools form hydrogen bonds to side chains or the main chain of the protein. Interactions for the main site of B3C (corresponding to site 1 in Fig. 4 ▶) are shown in Fig. 5 ▶. One carboxylate group interacts with a serine residue and the amide H atom of the protein backbone. The other carboxylate group interacts with the amino group of an asparagine through hydrogen bonding. Interactions of the amino group with the protein or other interactions of the carboxylate groups are also observed.

Restraints for the refinement were derived from the small-molecule crystal structures of I3C (Beck & Sheldrick, 2008 ▶) and B3C (Beck, Herbst-Irmer *et al.*, 2009 ▶). Model and restraints files for *REFMAC* (CIF format) and *SHELX* are available by email request from TB.

### 5.3. Radiation damage   

**Figure 6:**
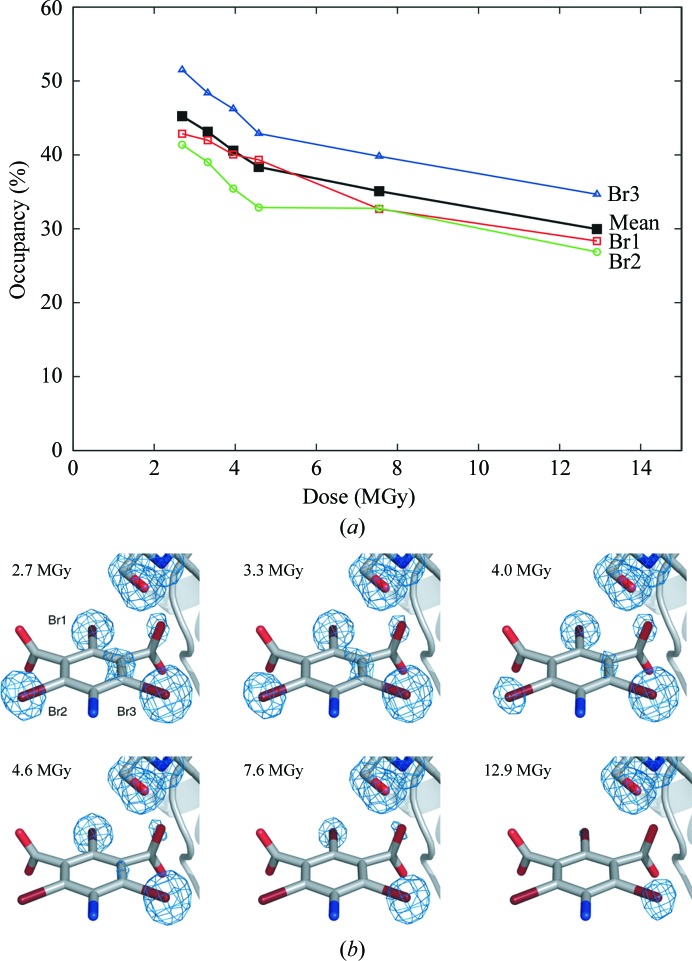
Radiation damage for B3C site 1 in proteinase K. The absorbed dose for the crystal is given in MGy (1 Gy = 1 J kg^−1^). (*a*) Refined occupancies (*SHELXL*) for the three Br atoms and the mean value (black) of site 1. The first data point is the occupancy after MAD data collection and a single burn and the subsequent five points show the change in occupancy with increasing dose (see §[Sec sec4.2]4.2). A drop in the refined occupancies of about 15% is observed after a dose of 12.9 MGy. (*b*) Electron density at site 1 contoured at 1σ. The serine residue is used as a reference since its density is not affected by irradiation. The density at the Br atoms clearly decreases with dose, but not all three Br atoms are affected equally.

The effect of irradiation is depicted in Fig. 6 ▶. Owing to changes in the experimental setup after the MAD experiment (the crystal-to-detector distance was changed from 160 to 200 mm and the crystal was re-centred after deicing), the occupancies from the refinement against peak2 (see above) deviate from the occupancies obtained from the radiation-damage experiments and are therefore not depicted in Fig. 6 ▶. A loss of about 15% in the occupancy of the bromine sites can be observed (Fig. 6 ▶ *a*) after the MAD experiment and six consecutive radiation-damage experiments (burn–collect). The program *RADDOSE* (Murray *et al.*, 2004 ▶) was used for dose calculations. Interestingly, not all bromine–carbon bonds suffered to the same extent on irradiation (Fig. 6 ▶ *b*). Although the bromine–carbon bonds were cleaved, the MAD experiment was still successful. Further MAD experiments with B3C will show whether radiation damage can actually obstruct structure solution with B3C.

Preliminary results from radiation-damage experiments with I3C (results not shown here) indicate that I3C is at least as susceptible as B3C to radiation and probably even more. Similar results have been reported for halogenated nucleotides (Oliéric *et al.*, 2009 ▶).

## 6. Conclusion   

B3C and I3C represent a novel class of compounds that show interaction with protein molecules. These sticky phasing tools may be utilized for experimental phasing. I3C is the compound of choice for in-house data collection since the I atoms give rise to a strong anomalous signal with Cu *K*α radiation (SAD or SIRAS phasing). If diffraction is too weak for in-house phasing, B3C is suitable for MAD data collection at a synchrotron beamline, taking advantage of the additional phase information from data collection at different wavelengths.

The fixed geometrical arrangement of the heavy atoms facilitates structure solution since the triangles can readily be identified in the heavy-atom substructure. A new version of *SHELXD* that takes this information into account is currently being tested. B3C and I3C could serve as test candidates for further investigations of the anisotropy of anomalous scattering. The molecular arrangement of the anomalous scatterers provides additional information for refinement of the parameters that describe the anisotropy of the anomalous scattering.

The effect of radiation damage on B3C and its phasing capabilities has been investigated. Although the bromine–carbon bond in B3C suffers considerably from radiolysis, a MAD experiment could still be carried out successfully. In order to take advantage of the radiolysis of the anomalous scatterers, it is recommended that the inflection data set is collected at the end of the MAD experiment. The lower occupancy of the bromine sites arising from radiation damage caused by previous data collection results in an increase in the dispersive signal. The possibility of carrying out radiation-induced phasing (RIP; Ravelli *et al.*, 2003 ▶) with the compounds presented here cannot be excluded, but was not further investigated within the scope of this study.

We find that B3C binds to the surface of the protein at the periphery. Interestingly, although several modes of hydrogen-bonding interactions have been observed for B3C and I3C, no aromatic interactions have been found in the crystals investigated to date. The bulky halogen atoms and the carboxylate groups arranged perpendicular to the benzene ring may hinder π–π interactions with aromatic side chains.

In this study, the phasing tools were incorporated by soaking. There is a recent example in which B3C was incorporated into protein crystals by means of cocrystallization, resulting in a high occupancy of the B3C site (Beck, de Cunha *et al.*, 2009 ▶). Currently, new phasing tools with different functional groups are being synthesized and tested. It has been noted previously that small molecules similar to B3C and I3C can promote crystal growth. A crystallization screen with a set of I3C/B3C-like molecules having different functional groups should shed some more light on this issue.

## Supplementary Material

PDB reference: proteinase K with B3C, 3gt3

PDB reference: proteinase K with I3C, 3gt4

## Figures and Tables

**Table 1:** Data-collection details for proteinase K with B3C and I3C Values in parentheses are for the highest resolution shell.

	B3C				
	Peak1	Peak2	High remote	Inflection point	I3C
Unit-cell parameters ()	*a* = *b* = 67.84, *c* = 101.77				*a* = *b* = 67.78, *c* = 101.84
Space group	*P*4_3_2_1_2				*P*4_3_2_1_2
Wavelength ()	0.9197	0.9202	0.9129	0.9204	1.5418
Photon energy (eV)	13480	13473	13580	13471	8042
Resolution ()	48.01.50 (1.601.50)	48.01.50 (1.601.50)	48.01.50 (1.601.50)	48.01.50 (1.601.50)	47.91.76 (1.861.76)
Total images	180	180	100	100	180
*R*_merge_ †	0.072 (0.23)	0.068 (0.28)	0.065 (0.23)	0.062 (0.22)	0.048 (0.13)
Completeness (%)	99.8 (99.6)	99.8 (99.2)	99.8 (99.4)	99.7 (99.2)	93.8 (62.0)
Multiplicity	14.1 (12.9)	14.1 (12.9)	7.9 (7.4)	7.8 (7.2)	10.14 (1.73)
*I*/(*I*)	27.3 (10.0)	24.9 (8.3)	22.0 (7.6)	23.0 (7.8)	33.8 (8.3)

† *R*_merge_ = 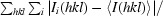 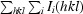.

**Table 2:** Refinement details

	Proteinase K with B3C	Proteinase K with I3C
PDB code	3gt3	3gt4
Data set used for refinement	Peak2	
Resolution ()	48.01.50	47.91.76
No. of reflections	38826	22770
*R*_cryst_/*R*_free_ (%)	14.3/18.4	14.0/19.1
No. of protein atoms	2045	2001
No. of ligand/ions atoms	74	53
No. of water atoms	417	343
*B* factors (^2^)		
Protein	11.04	7.91
Ligands	15.77	15.80
Waters	28.32	22.70
R.m.s. deviations		
Bond lengths ()	0.009	0.007
Angle distances ()	0.024	0.023
Ramachandran plot		
Favoured (%)	97.8	97.8
Disallowed (%)	0	0

**Table 3:** Comparison of the phasing statistics for the B3C and I3C data sets The mean phase error and mean map correlation coefficient compare the first experimental map obtained after density modification with *SHELXE* with the final refined map (*SHELXL*). MAD phasing with B3C results in better starting phases: the mean phase error is lower, the mean map correlation coefficient is higher and more residues can be traced compared with the SAD data set.

	B3C (MAD)	I3C (SAD)
*SHELXD* CC/CC(weak)	46.6/37.6	52.0/32.5
Mean phase error ()	31.2	36.9
Mean map CC	0.861	0.786
Residues traced (*ARP*/*wARP*)	272/279 (97.5%)	267/279 (95.7%)

## Footnotes

1 Until the year 2000, more structures were solved using MIR or SIR (single isomorphous replacement) than with MAD/SAD techniques (a total of 394 structures solved using MIR/SIR compared with 109 solved using MAD/SAD were deposited between 1995 and 2000). However, since then more structures have been solved using SAD or MAD methods (a total of 5760 solved using MAD/SAD compared with 1101 solved using MIR/SIR have been deposited since 2000) and the fraction of structures being solved using MAD/SAD is still increasing. PDB statistics were kindly provided by Clemens Vonrhein. PDB entries with REMARK 200 set to NULL or OTHER were ignored. Date of PDB analysis: June 10, 2009.

2 The p*K*_a_ values of the two carboxyl groups in B3C could not be determined experimentally by potentiometric titration (probably owing to the fact that the molecule forms a zwitterion in solution); the p*K*_a_ values of the nonbrominated compound have not been reported in the literature. To obtain the stock solution, the compound cannot be dissolved directly in water since it has a poor solubility. After adding double equimolar amounts of base, the two carboxyl groups are mostly deprotonated, producing a salt with high solubility. The pH of the stock solution is usually in the range 7–8.

3 The atomic scattering factor of bromine was adjusted to the given wavelength (DISP *f*′ *f*′′ with *f*′ = −8.7 and *f*′′ = 3.47, experimental values).
